# A Comparative Study of Optical Sensing Methods for Colourimetric Bio/Chemical Detection: Cost, Scale, and Performance

**DOI:** 10.3390/s25133850

**Published:** 2025-06-20

**Authors:** Cormac D. Fay, Liang Wu, Isabel M. Perez de Vargas Sansalvador

**Affiliations:** 1 School of Medical, Indigenous and Health Sciences, Faculty of Science, Medicine and Health, University of Wollongong, Wollongong, NSW 2522, Australia; 2 Melbourne Centre for Nanofabrication (MCN), Department of Materials Science and Engineering, Monash University, 22 Alliance Lane, Clayton, VIC 3168, Australia; liang.wu@monash.edu; 3 Electronic and Chemical Sensing Solutions (ECsens), Department of Analytical Chemistry, University of Granada, 18071 Granada, Spain; isabelpdv@ugr.es; 4 Unit of Excellence in Chemistry, Biomedicine and the Environment of the University of Granada, 18071 Granada, Spain

**Keywords:** colourimetric, bio/chemical, sensing, comparison, PEDD, imaging, LED

## Abstract

**Highlights:**

**What are the main findings?**

- Robust Comparison: We evaluated three optical sensing approaches for bio/chemical determination using a pH indicator.
- Superior Performance: The PEDD method showed superior resolution, accuracy, sensitivity, and detection limit results.
- Ratiometric Analysis: LED photometry outperformed spectrophotometry and imaging in all key metrics.
- Versatile Solution: The PEDD approach emerged as a versatile and high-performance solution for precise sensing.
- Valuable Insights: Our comprehensive evaluation offers valuable insights for optimal optical sensing methods.

**What is the implication of the main finding?**

- Enables low-cost, high-performance sensing for industrial use.
- Facilitates scale-up for decentralised and autonomous systems.
- Reduces dependence on complex lab-based instrumentation.
- Informs method selection for application-specific needs.
- Accelerates translation of sensing research into practice.
- Highlights tradeoffs between accuracy, cost, and complexity.

**Abstract:**

This study provides a detailed comparison of three optical sensing approaches for colourimetric bio/chemical detection, focusing on cost, scalability, and performance. We examine laboratory-grade spectrophotometry, portable camera-based imaging, and low-cost LED photometry using Paired Emitter–Detector Diode (PEDD) charge–discharge methodology. Our findings reveal that while the LED-based PEDD system outperforms the other two methods in key sensory metrics—such as sensitivity, resolution, and limit of detection—its cost-effectiveness and scalability make it a promising solution for widespread industrial and field applications. Compared to the spectrophotometer, the LED/PEDD approach demonstrates improvements in measurement range (×16.39), dynamic range (×147.06), accuracy (×1.79), and sensitivity (×107.53). The results highlight the potential for industrial-scale adoption of LED photometry, especially for cost-effective applications in bio/chemical sensing sectors.

## 1. Introduction

In recent years, the importance of collecting accurate bio/chemical information has grown dramatically in the scientific [1], environmental [2], health [3,4], and industrial domains [5]. Rapid and reliable quantification of analytes is essential for a wide range of applications, from environmental monitoring to biomedical diagnostics [6,7,8], and it is becoming increasingly important in industrial sectors such as food safety, pharmaceutical processing, wastewater treatment, and manufacturing compliance. Currently, the model for determining bio/chemical markers from either the environment or personal health typically involves a number of operations, i.e., sample collection, transport, storage, and analysis in centralised laboratories by sophisticated instrumentation and by highly trained personnel, in addition to data processing, input, and delivery [9,10]. There are good reasons for using this model, which includes its acceptance and high-quality assurances critical for areas such as those under legislative compliance and others prone to legal disputes. As the demand for this information increases, this model is not inherently scalable and critically requires the development of low-cost and autonomous systems to meet the requirements of today [11,12,13]. This was captured by Prof. Royce Murray (Editor for Analytical Chemistry), who once commented that “A ‘Grand Challenge’ posed for analytical chemistry is to develop a capability for sampling and monitoring air, water and soil much more extensively and frequently than is now possible…Such goals will require improvements in sampling methodology and in techniques for remote measurements, as well as approaches that greatly lower per-sample and per-measurement costs” [14].

This overarching challenge necessitates the further fusion of technology with well-proven and reliable chemo-responsive materials for bio/chemical sensing [15]. Achieving bio/chemical information in a cost-effective and continuous manner, however, still remains a formidable challenge for the sensing research community [7,14,16]. While established responsive materials exist for sensing purposes, the fusion of technology is essential to facilitate the translation of proposed bio/chemical sensors into reliable, cost-effective, scalable solutions for industry adoption and distribution [17]. One solution is through optical sensing, which has emerged as one of the most reliable, cost-effective, and rapid methods for obtaining bio/chemical information [2,8]. In terms of the complementary technology [18], diagnostic systems can be broadly categorised into three approaches, i.e., (a) laboratory via spectrophotometers, (b) portable such as smartphones, and (c) autonomous devices involving selective components—e.g., classically via photodiodes [19].

The field of optical sensing has witnessed significant advancements, with spectrophotometry [20,21], LED photometry (Paired Emitter–Detector Diode or PEDD for short) [22,23,24,25], and imaging [26,27,28,29] emerging as prominent methods for colourimetric measurements. Extensive reviews of the literature reveal commendable achievements in each approach, showcasing their unique strengths and applications for, e.g., environmental [15,30,31,32,33] and health/wearable applications [34,35,36,37,38]. From the precision of spectrophotometric analyses to the sensitivity of the PEDD approach and the ubiquitous richness of imaging techniques, e.g., via phone cameras [27,39,40], these methods have individually contributed to the expanding landscape of optical sensing.

Despite the individual successes of these approaches, a comprehensive comparison is notably absent from the existing literature. While certain scenarios may dictate a clear preference for one method over the others, the absence of a comprehensive comparative study leaves a critical gap in informing/guiding scholars and industry professionals towards an informed choice. Each approach comes with its own set of advantages and disadvantages, making it crucial to evaluate their performance across multiple criteria to determine the most suitable option for a given application.

In this paper, we bridge this gap by presenting a thorough and systematic comparison of spectrophotometry, LED photometry (PEDD), and imaging for pH measurements via colourimetric means. Our study goes beyond singular evaluations, encompassing a range of sensory characteristics, including resolution, accuracy, sensitivity, and limit of detection. Through a series of experiments and analyses, we provide a nuanced understanding of the strengths and limitations of each approach, offering valuable insights for researchers, scholars, and industry practitioners seeking the most fitting optical sensing method for their specific colourimetric measurement needs. Particular attention is given to the practical implications of each method for industrial-scale real-world deployment, highlighting considerations such as scalability, operational simplicity, robustness, and total cost. These criteria are critical for the widespread adoption of optical sensing systems in distributed or autonomous monitoring architectures, including those deployed in resource-limited, harsh, or regulated environments.

## 2. Materials and Methods

### 2.1. Experimental Overview

All experiments were conducted in a certified analytical chemistry laboratory (NATA-accredited) to ensure high standards of measurement accuracy and repeatability. The laboratory environment was maintained at a constant temperature of approximately 22 °C with controlled humidity levels (relative humidity maintained at approximately 50%) throughout the experimental period. The study involved the preparation of bromocresol green (BCG) solutions across a range of pH values and their characterisation using three optical sensing approaches: spectrophotometry, LED-based photometric detection, and imaging analysis. This section outlines the detailed methodologies employed, including solution preparation, reference measurements, instrumentation, and the procedures for comparative evaluation.

### 2.2. Sample Preparation

A 50 μM pH dye stock solution was prepared by dissolving 9 mg (measured using a Lennox Adventurer OHAUS alance) of bromocresol green (BCG) powder (Sigma Aldrich, Sydney, NSW, Australia, 114367-5G) into 250 mL of ultrapure water (Millipore Milli-Q, Sydney, NSW, Australia). BCG was selected because of its large molar extinction coefficient and spectral distribution similar to the CIE 1931 RGB/XYZ waveforms. Volumes of 100 mL of 0.1 M HCl and 100 mL of 0.1 M KOH were prepared from 37% HCl (Scharlau, Sydney, NSW, Australia, AC0741) and 8 M KOH solution (P4494, Sigma Aldrich, Sydney, NSW, Australia), respectively. Solutions spanning pH 2–8 were prepared through a controlled titration process involving precise additions of HCl, ultrapure water, and KOH, yielding 10 mL per sample stored in 50 mL polypropylene vials. Each solution was thoroughly mixed and allowed to stabilise for 5 min prior to further use. A volume of 10 mL of the BCG stock solution was added to each prepared solution, ensuring a consistent concentration of 25 µM BCG in each solution. A volume of 2 mL from each final solution was then transferred to cuvettes for subsequent optical analysis.

### 2.3. Reference Measurements

A pH meter (Cyberscan PCD 6500, Instrument Choice, Sydney, NSW, Australia) and probe (MI-415-2, Microelectrodes, Inc., Adelab Scientific, Sydney, NSW, Australia) were calibrated with pH buffers (4 and 7) in accordance with manufacturer instructions. Each of the solutions was then measured in triplicate, and the electrode was rinsed with Milli-Q water and gently dried between measurements to avoid cross-contamination. Following this, each solution’s absorption spectrum was determined using a spectrophotometer (Cary 50 UV-Vis, Varian, Sydney, NSW, Australia). Each measurement was performed from 350 nm to 750 nm in 1 nm increments in triplicate, and each vial was shaken sufficiently to ensure a homogeneous solution. The Cary WinUV software offered a selection of 5 scan rates from ‘Slowest’ to ‘Fastest’. For this study, the ‘Medium’ rate was selected. Based on available data from the manufacturer, the maximum scan rate is 24,000 nm per minute. The available specifications also state that the ‘UV-Vis limiting resolution’ (nm) is ±1.5 nm.

### 2.4. Optical Analysis Set Up

**Figure 1:**
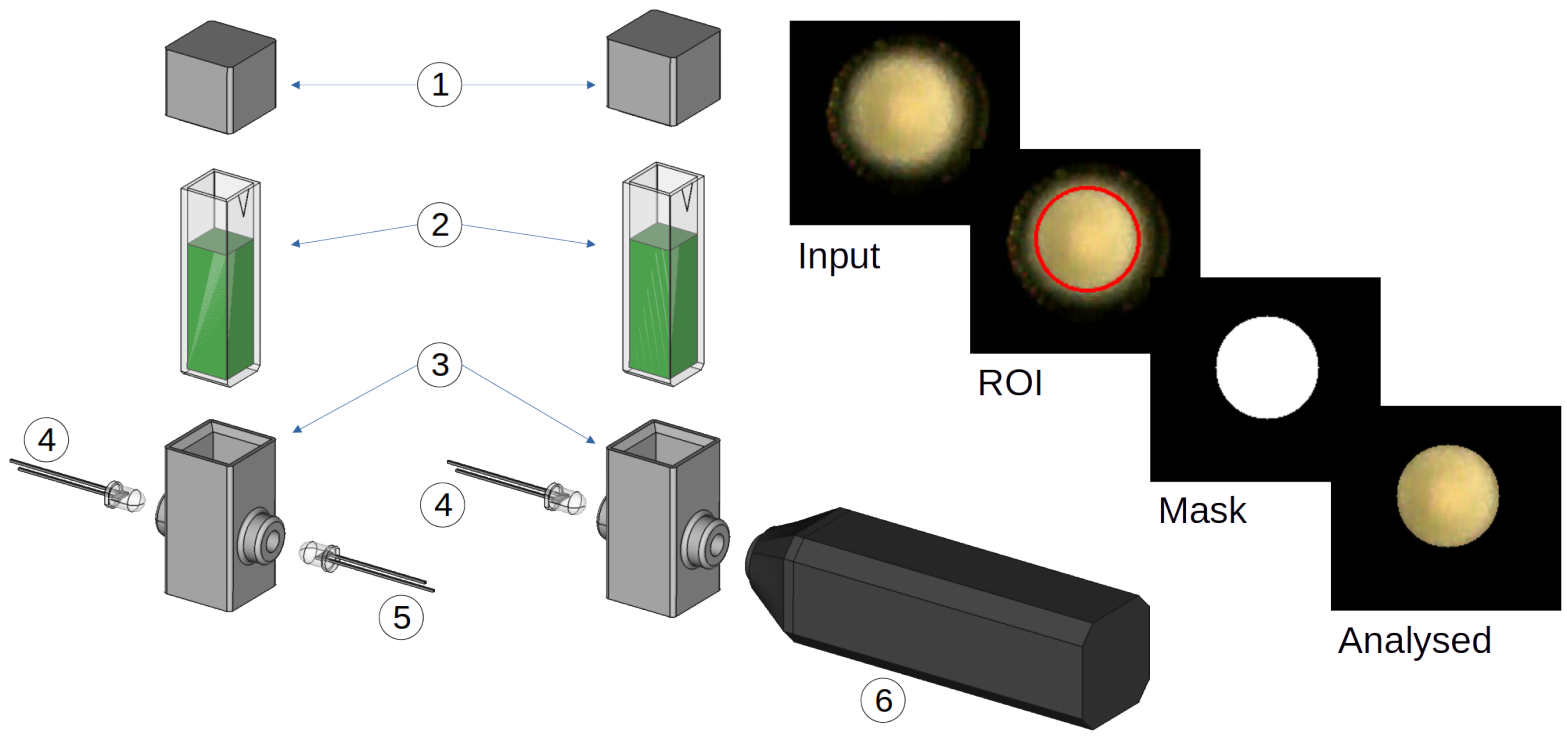
An exploded view of the optical components setup for the LED (**left**) and imaging analysis (**centre**). A customised 3D-printed cuvette holder equipped with two 5 mm mounts (emitter and detector) for absorbance mesurements–detector LED and camera are interchangeable. An example of the image processing stages are provided (**right**). Labelled components: (1) cuvette holder top; (2) 3 mL cuvette; (3) cuvette holder base; (4) emitter LED; (5) detector LED; (6) camera.

**Figure 2:**
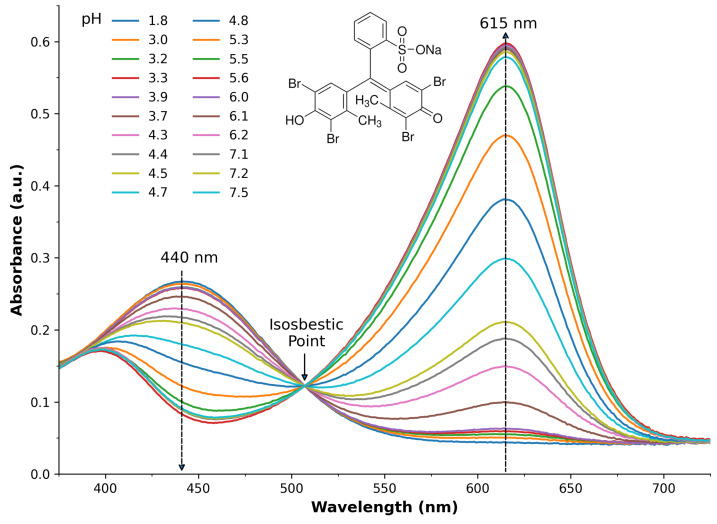
Spectral measurements of the samples shown in the visible range of 400–700 nm. The two most prominent peaks are identified via the vertical dashed lines at 440 nm and 615 nm ($\lambda_{max}$), indicating increasing pH. **Top centre**: Inset image shows the structure of the BCG dye for reference. **Top left**: Legend shows the measured pH value.

Figure 1 presents the optical setup for analysis via the LED and imaging (camera) approaches. Here, a cuvette holder was designed using CAD software (FreeCAD v1.0, 39109) and fabricated via a 3D printer (Dimension SST 768, Stratasys, Melbourne, Australia). The arrangement was chosen to more accurately represent the same conditions used by the spectrophotometer when analysing the samples within a 3 mL cuvette. For absorbance measurements, the holder includes two mounting points to securely hold two 5 mm LEDs facing each other, with space for a cuvette placed in between. For the imaging analysis, and to provide consistency for comparative purposes, the detector LED was replaced with a small camera, with sample images provided in the figure (processing discussed later). The figure below presents the holder in grey for visual clarity, whereas the fabricated holder was printed in black ABS to reduce specular reflections during analysis. A cuvette top was also provided to help isolate light from external sources during analysis. To further help with isolation from possible external light interferences, the setup was placed within a light-enclosed box. The design allowed easy access to interchange the 3 mL cuvettes holding each prepared solution, as shown in Figure 2.

### 2.5. LED Measurements (PEDD Approach)

Figure 1 (left) presents the physical arrangement and the required components for LED analysis. An emitter and detector LED (Toyoda Gosei E1L53-AW0C2, RS Components AU Distributor, Sydney, NSW, Australia) was securely placed facing each other in the 5 mm LED mounts as designed. A forward voltage of 2.3532 V was applied to the emitter LED (powered by TTi EL302TV and measured with a TTi 1604). For the detector LED using the charge/discharge approach, this process is outlined in more detail in previous studies [41]. Briefly, the detector LED was connected to a microcontroller (MSP430 F449) with its cathode connected to an Input–Output (IO) pin (Port A, Pin 7) and its anode to a GND pin. Listing 1 presents the controlling C code for performing the PEDD measurements using bit registers and assembly operators for efficient operation. Here, the detector LED was initially charged by the IO for 100 µs by setting the IO pin to output mode and the pin to 3.3 V (denoted as high). The 100 µs charge time was determined heuristically to ensure full charge. After this, the LED was allowed to discharge by switching the IO port to input mode. During this time, the voltage of the LED was monitored via the IO pin (input mode), where two software registers were used to count the samples. When the LED voltage was above the logic threshold, the ‘highCounter’ register was incremented and similarly the ‘lowCounter’ for when below. The reason for providing a balanced counter was to avoid errors in the timing aspects, as discovered in previous work [42]. The data were finally reported to the user screen via the MSP430 and a USB cable, where they were recorded to a text file for later analysis.

**Listing 1.** PEDD charge/discharge firmware.

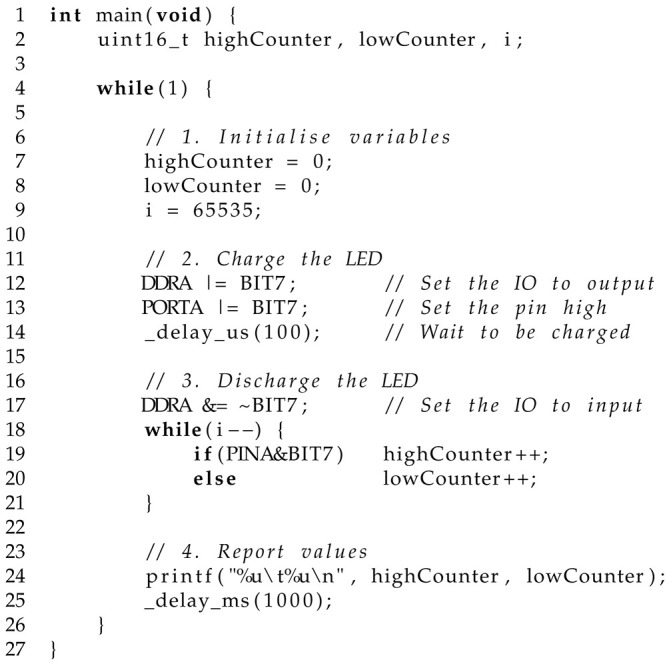



### 2.6. Imaging Analysis

Figure 1 (centre) presents the physical setup for analysing the solutions using an imaging device. The setup was the same as with the LED approach, with the exception that a camera (ZTV ZT830T) took the place of the detector LED. This allowed for a common approach in measurement and therefore provided a basis for comparison. The forward voltage of the emitter LED (2.3532 V) was chosen in order to achieve an emission intensity that was balanced between the camera’s lower visible detection limit and saturation, i.e., the blooming effect.

For image processing, the camera was set to captured video with the following parameters:
Spatial Resolution:180 × 160 (W × H);Frame rate:15 fps;Colour space:sRGB;Codec:24 bits RGB (RV24)-Uncompressed;Container:Audio Video Interleave (AVI).

A video of the solutions was recorded for 20 s, followed by 10 s to allow removal and interchange with subsequent samples. The video was saved in uncompressed format to avoid artefacts arising from compression effects. While the majority of the research field typically relies on static snapshots, it is important to note that in industrial applications, such as assembly line analysis, rapid detection must occur in quick succession to meet operational demands. Additionally, since the PEDD takes multiple temporal measurements, it was considered prudent to adopt the same approach here for comparative purposes.

Figure 1 (right) presents an example image of the image processing taking place during analysis—see images arranged from top left to bottom right in a diagonal arrangement. Due to the physical constraints, it was not necessary to develop segmentation algorithms to isolate the area of interest. In place, a circular region of interest (ROI) was determined heuristically (5000 pixels)—see the red circle in the ROI image. This allowed for the removal of the surrounding pixels that are a result of reflections from the clear cuvettes that may interfere with analysis. A mask area was subsequently created (full white circle) that identified the pixels to keep. When applied to the original image/frame (binary AND operation), the surrounding pixels were removed with central pixels retained. These retaining pixels were processed by calculating the average of each frame’s sRGB channel representing a measurement for that frame. The sRGB colour space was chosen due to its compatibility with BCG on the CIE xy chromaticity diagram, as determined previously when comparing 68 RGB colour spaces [43].

### 2.7. Comparative Analysis of the Three Approaches

Four key characteristics critical to describing sensor performance and that formed the basis for comparison were identified as range/resolution, accuracy, sensitivity, and limit of detection (LOD). Based on previous works in the literature, pH indicators often exhibit a sigmoid shape in their absorbance outside of their linear range (typically 1–2) pH units. As a result, the Boltzmann model shown in Equation (1) was prepared for fitting of the responses. Additionally, the derivative of this response can yield an estimate of the dye’s p$K_{a}$ [44,45], which is presented in Equation (2). The Levenberg–Marquardt [46,47] algorithm was used for regression analysis as part of the scipy [48] python package. Other libraries/packages were used as follows: OpenCV [49] for image/video analysis, sqlite3 [50] for data storage, numpy [51] for general data handling/preparation, and matplotlib [52] for generating the plots in this manuscript.

(1) $$f\left(x\right)=\frac{y_{1}-y_{0}}{1+e^{(x-x_{0})/dx}}+y_{0}$$

(2) $$f^{\prime }\left(x\right)=\frac{\left(y_{1}-y_{0}\right)}{dx}\times \frac{e^{(x-x_{0})/dx}}{{\left(1+e^{(x-x_{0})/dx}\right)}^{2}}$$

where
$y_{1}$:Final value;$y_{0}$:Initial value;$x_{0}$:Centre;dx:Exponential constant.

## 3. Results and Discussion

### 3.1. Spectrophotometry

Figure 2 presents the spectra of the samples when analysed by the spectrophotometer. Two prominent peaks are observable at 440 nm and 615 nm. As the pH increased, the prominent waveform transitioned from 440 nm to 615 nm, causing a colour change from yellow to blue, respectively, visible in the cuvette solutions shown in Figure 2. The isosbestic point was also detected at 515 nm. All of these characteristics are in line with previously published data using BCG [43,53].

**Figure 3:**
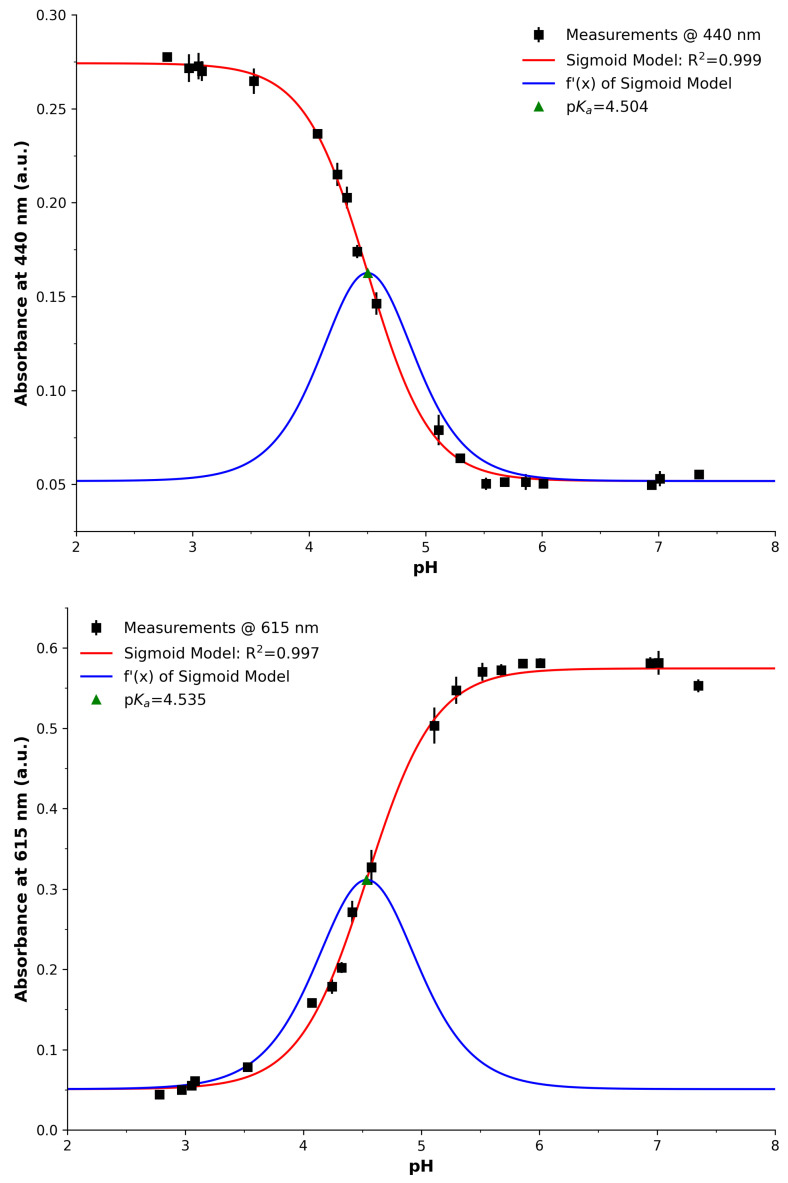
Spectral absorbance at 440 nm (**top**) and 615 nm (**bottom**); black squares represent the average of three measurements, with error bars as the standard deviation. The red line is an excellent sigmoid model fit to the data: R^2^ > 0.997. The blue line represents the derivative of the sigmoid model, with the peak estimating the p$K_{a}$ at 4.504 (440 nm) and 4.535 (615 nm).

Figure 3 presents the absorbance values at 440 nm (left) and 615 nm (right) that were extracted from Figure 2 and plotted as a function of measured pH. The black squares represent the average across the three measurements, with the error bars as the standard deviation. The red line represents an excellent sigmoid fit to the data points with R^2^ = 0.997, and the optimised parameters are listed in Table 1 (Band 2). In order to estimate the p$K_{a}$ of the dye, the derivative of the fitted model was calculated using Equation (2) and shown in the respective sub-figures as the blue lines, with its peak determined to represent the point of maximum transition between the acid and base form. The p$K_{a}$s were estimated at 4.504 (440 nm) and 4.535 (615 nm), which slightly differ from the accepted value of 4.74 at room temperature [53] by 0.236 and 0.205, respectively. This is a very close approximation and in good agreement with previous reports in the literature, e.g., by O’Toole et al. [45], who estimated the p$K_{a}$ of BCG via PEDD to be 4.5.

### 3.2. LED Photometry

**Figure 4:**
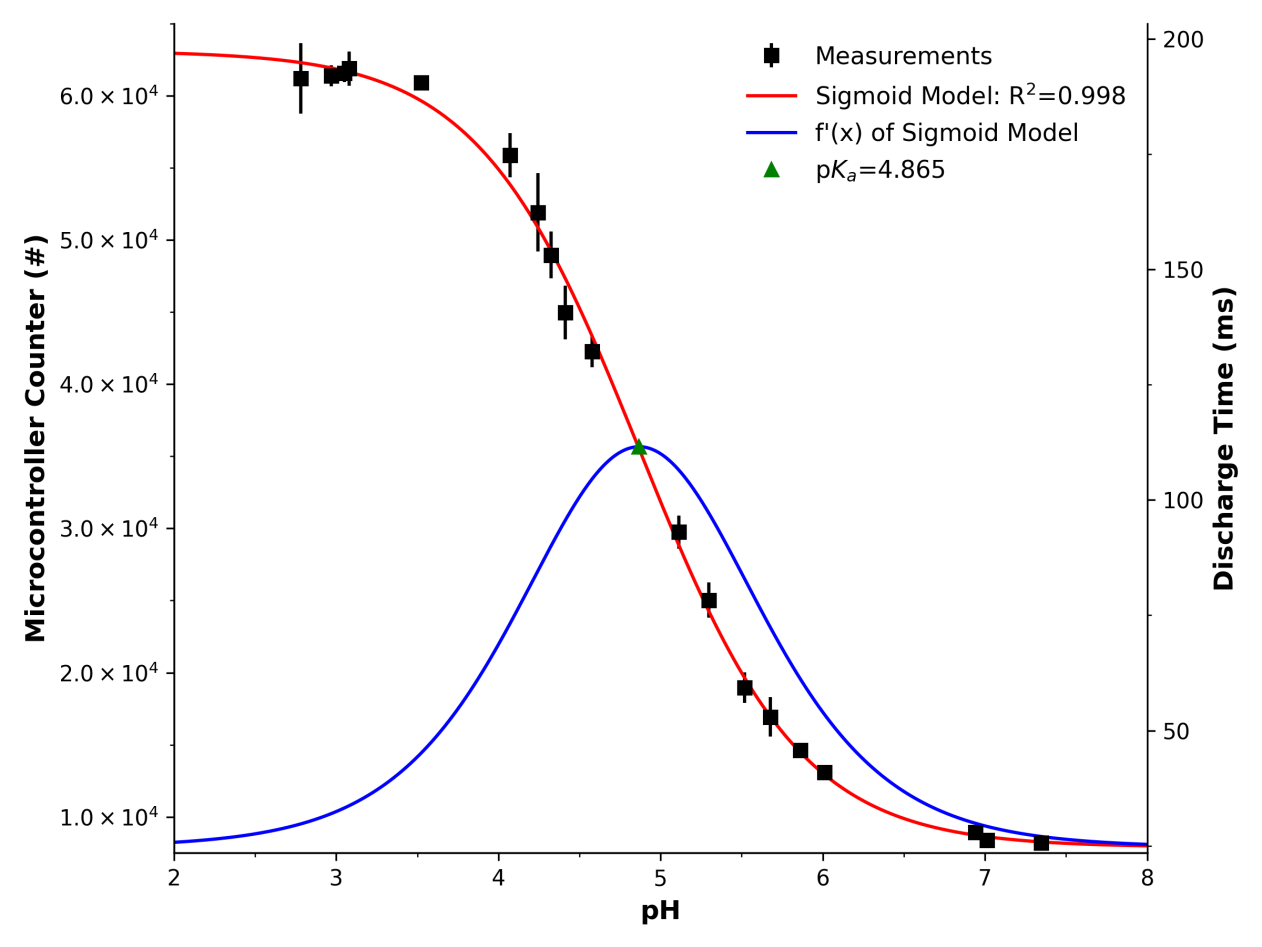
LED absorbance (PEDD measurements). Average values (black squares) and error bars (standard deviation) of three successive measurements. Red line is an excellent sigmoid model fit to the data (R^2^ = 0.998); see Equation (1). Blue line represents the derivative of the sigmoid model, see Equation (2), with the peak estimating the p$K_{a}$ at 4.865.

Figure 4 presents the response of the PEDD measurement as a function of measured pH. The vertical axes represents the duration required for the detector LED’s internal capacitance to discharge. This is represented in two ways, i.e., the software counter outlined in Listing 1 (left vertical axis) and the time taken to discharge (right vertical axis). The measurements were made in triplicate, and their average values are presented as black square markers, with the standard deviation shown as error bars. Upon first analysis, one can see that the trend decreased with increasing pH and also exhibited a sigmoid shape. This was confirmed by the fitting of the model in Equation (1), resulting in an excellent fit of R^2^ = 0.998, with optimised parameters provided in Table 1 for reference.

An estimation of the p$K_{a}$ was established via a derivative of the sigmoid model (Equation (2)) and appears as the blue line in Figure 4. Its peak was calculated and established as an estimation of the p$K_{a}$ value at 4.865. This is a very close approximation of the p$K_{a}$ value of BCG, with a slight deviation of 0.125 from the established value of 4.74 at room temperature [53]. It is in excellent agreement with the reported p$K_{a}$ of 4.85 by Diamond et al. [54], who also studied BCG using the PEDD technique. These accounts demonstrate that there is high confidence in the PEDD system and analysis used in this study and at an excellent level for comparing optical sensing platforms.

### 3.3. Imaging Analysis

**Figure 5:**
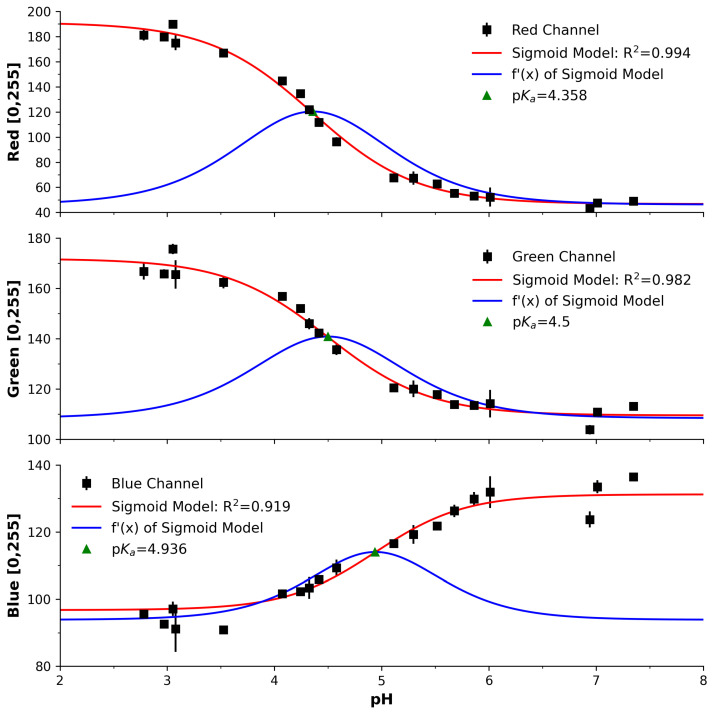
Results of the imaging analysis, i.e., response of the camera’s RGB channels to sample’s pH; Red (**top**) Green (**middle**), Blue (**bottom**). Black square markers represent the average across repeated measurements with error bars as the standard deviation. Red line in each represents good sigmoid fits of R^2^ = 0.994 (Red Channel), R^2^ = 0.982 (Green Channel), R^2^ = 0.919 (Blue Channel).

Figure 5 presents the response of the three imaging channels (Red, Green, and Blue) after processing of the captured videos in sRGB colour space. The y axis represents unitless pixel intensities on an 8-bit scale (0–255). The black squares represent the average across successive measurements, with the red lines demonstrating excellent sigmoidal fits (R^2^: 0.982 to 0.994) as per Equation (1). In a similar manner to the previous spectra and PEDD analysis, the derivative of the model is presented as the blue lines, with its peak estimating the p$K_{a}$. While the red channel offers a larger range, the green channel offers the most agreeable p$K_{a}$ across the channels to the accepted value of 4.74. It appears that each channel may offer advantages and must, therefore, be subject to comparison. As a result, our next sections will investigate each channel as comparative elements of the imaging approach.

### 3.4. Comparison

For comparing the spectrophotometer, PEDD, and imaging approaches in terms of various sensing factors/characteristics, we considered the information generated previously in addition to the listed data provided by the optimised fitted parameters and characteristics summarised in Table 1. Considering that all samples were analysed in the same manner (path length and optically) by all three devices, it stands to reason that the following comparisons are comparable.

#### 3.4.1. Dynamic Range and Resolution

For clarity, it is important to first define the difference between dynamic range and detection range. The dynamic range of the analyte (via a colourimetric reagent in this case) refers to the span of values over which it can accurately represent/indicate changes. In the context of a colourimetric reagent used with a sensor device, the dynamic range typically represents the pH range over which the reagent changes colour in response to varying acidity or alkalinity levels. On the other hand, the detection range of a sensor device/instrument specifies the range of values within which the sensor can reliably measure a parameter—we denote this as the sensor’s full scale deflection (FSD). Ideally, the reagent’s dynamic range should lie entirely within the sensor’s detection range to ensure accurate and meaningful measurements. In the context of sensors measuring bio/chemical targets, such as pH in this work, a wide dynamic range is crucial for accommodating variations in analyte concentrations that can occur in different samples. The dynamic range determines the span between the lowest and highest concentrations that a sensor can reliably detect. This was determined by $|y_{1}-y_{0}|$ of the optimised parameter fittings in Table 1. Considering that each device performed measurements with different resolutions and steps (increments), it was necessary to normalise their data for comparative purposes.

Herein, resolution refers to the smallest measurable change that the sensor can reliably distinguish, reflecting its ability to detect subtle variations within the measurement range. It is important to distinguish this from instrument resolution, which is the minimum increment the device hardware can report—denoted as Unit Step in Table 1. Together, these values provide a fuller picture of both the device’s and the system’s practical capability.

Each device incremental step was determined through the lower (0) and upper limits, which were determined through the bit depth of the PEDD (16-bit) and imaging device (3 × 8 bits). For the spectrophotometer, this was established from the manufactuer’s advertised capabilities, i.e., a range of 0–4 absorbance (a.u.) with three decimal places of precision (unit step). The total increments allowed for normalisation and the features in Table 1 denoted as the ‘Increments’ field. The resolution of the instrument was therefore established by dividing the instrument’s dynamic range by the number of increments.

**Figure 6:**
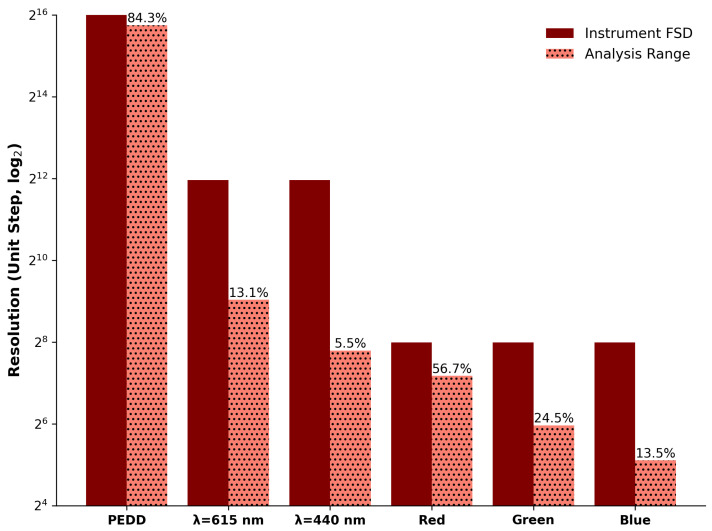
Comparison of all approaches based on resolution and range ranked from highest to lowest (**left** to **right**). The devices’ full scale deflection (FSD) values are shown in maroon colour (**left** bars). The normalised analysis range of the FSD is shown in salmon colour (**right** bars, dotted), with their percentages (placed above for clarity) representing the proportion of the dynamic range to the detection range.

Figure 6 presents a comparison of all the approaches’ detection (solid bars) and dynamic (dotted bars) ranges with the numeric proportion (%) provided above for visual reference. It can be seen that the PEDD was the most optimised for analysing the samples in this study. This was not only due to the first ranking but by a factor of 100 times that of the second ranked device (spectra at 615 nm). The capabilities of the PEDD approach are understandable, as one can tune the device to suit the analyte under investigation, whereas the spectrophotometer and imaging device are fixed with the manufacturers’ settings. This highlights the capability and flexibility of the PEDD approach, which demonstrates excellence for this comparative parameter. It must be mentioned that it may not hold strength against spectrophotometers and/or imaging devices that can analyse other colourimetric reagents across the visible spectrum. This would be an excellent study to perform, but it is outside the scope of this work.

In terms of the devices other than the PEDD, the spectrophotometer clearly outperformed the imaging by a factor of 3.6 when comparing their two best-performing options, i.e., λ = 615 nm against the imaging’s Red Channel. It is worth considering that we are comparing a benchtop instrument against imaging technology, which is almost ubiquitous as the standard features on mobile devices. As portable devices, the reduction in resolution may be acceptable, yet one must also consider other imaging factors that may affect its performance, e.g., colour space, bit depth, lighting conditions, and others.

In addressing the integral relationship between the dynamic range and the detection range, it is vital to emphasise that while the dynamic range specifies the operational span within which a sensor can function accurately, the detection range particularly defines the actual limits within which the sensor can detect the analyte under specific conditions. Our study meticulously characterises the dynamic range through the span $|y_{1}-y_{0}|$ derived from optimised parameter fittings, which effectively maps out the sensor’s ability to capture and quantify signal variations corresponding to different analyte concentrations. Moreover, the detection range is critically dependent on both the intrinsic properties of the sensor and the dynamic range it offers. A broader dynamic range enables a wider detection range, allowing for more flexible and accurate analyte measurement across diverse sample conditions. For instance, the PEDD’s enhanced dynamic range allows for superior detection capabilities in our tests, clearly highlighting the practical benefit of such optimisation in real-world applications where variable analyte concentrations are a norm rather than an exception. Our findings underscore the importance of selecting approaches with appropriately matched dynamic and detection ranges to the intended application to ensure both reliability and accuracy in measurements. Future investigations could explore how modifications to sensor design or operational parameters could further enhance the alignment between these ranges, potentially broadening the utility of the sensors across different analytical scenarios.

#### 3.4.2. Accuracy: p$K_{a}$ Estimation

Accuracy is paramount for sensors, as it ensures that measurements closely reflect the true value, while precision ensures that repeated measurements yield consistent results. Both qualities are essential for generating trustworthy and reliable data in sensing applications. In applications such as bio/chemical sensing, accurate sensor readings are essential for making informed decisions, whether in environmental monitoring, biomedical diagnostics, or industrial processes. High accuracy contributes to the credibility and utility of sensor data, influencing the effectiveness and success of various scientific and technological endeavours. To investigate a comparison of the devices on the basis of accuracy, we used the accepted value of the dye’s p$K_{a}$ (4.74 [53]) as the prime indicator.

Estimating the p$K_{a}$ of a chemical indicator is crucial, as it indicates the pH range over which the indicator undergoes a significant colour change. Selecting an indicator with an appropriate p$K_{a}$ is essential for ensuring optimal sensitivity within the desired pH range of a specific application. Additionally, accurate knowledge of the p$K_{a}$ is vital for understanding the sensory characteristics of the indicator, such as dynamic range and resolution, which directly impact the reliability and precision of pH measurements. It was therefore important to examine which device (and options thereof) was capable of p$K_{a}$ estimation most accurately.

**Figure 7:**
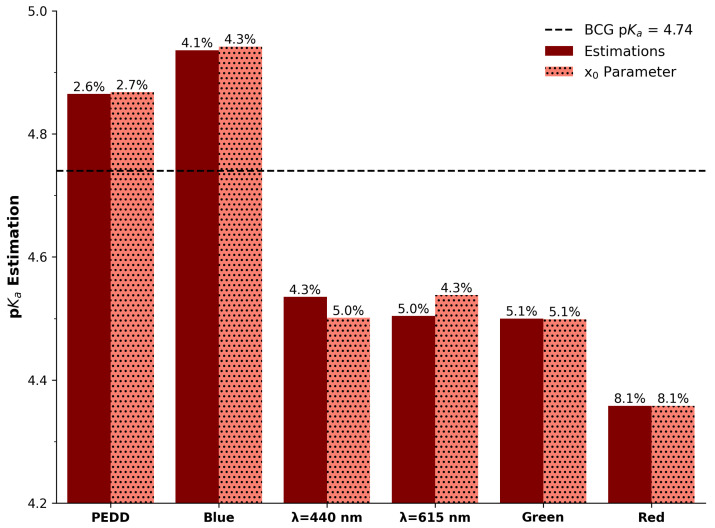
Estimation of the p$K_{a}$ of all approaches (**left** bars, maroon colour) ranked from left to right in terms of accuracy to the accepted value of 4.74 (dashed line). Estimations using the optimised parameter x_0_ (**right** bars, salmon colour, dotted). Numbers above the bars represent the percentage relative error to the accepted value of 4.74.

The estimated p$K_{a}$ values of all devices are summarised in Table 1 and provided visually in Figure 7. These are, on average, good approximations to the reference/established value of 4.74 at room temperature [53]. Curiously, the x_0_ parameter also corresponds with the estimated p$K_{a}$s and is largely in line with the differential-peak approach, which demonstrates that the Boltzmann fit is an excellent option for such analysis. For comparative purposes across the devices, the percentage relative error (%RE) was calculated using Equation (3) and relative to the reference p$K_{a}$ of 4.74. It is clear that the PEDD approach is in most agreement with the reference value, and the red imaging channel is the least in agreement. The remaining options are relatively comparable.

It is not exactly clear why the PEDD offers the best agreement to the accepted value of 4.74 over the others, but it may be due to the increased resolution of the device. Conversely, while the Red Channel of the imaging device offered good resolution, which is understandable due to the prominent peak in the red region of Figure 2, it offered the least favourable agreement to the p$K_{a}$. This does not, however, support the correlation of resolution and p$K_{a}$ estimation accuracy. Notwithstanding, overall and from a sensory perspective, it is clear that the ability to accurately detect aspects of interest from colourimetric reagents is critical and reflective of the performance of the optical interrogating device. As p$K_{a}$ is an excellent example of this, the PEDD approach offers the best performance in this regard, outperforming the camera (Blue) and the spectrophotometer (440 nm) by a factor of 1.59 and 1.67, respectively.

(3) $$\%RE=\frac{|pK_{a}\left(Ref\right)-pK_{a}\left(Estimated\right)|}{pK_{a}\left(Ref\right)}\times 100$$

#### 3.4.3. Sensitivity

In bio/chemical sensing, sensitivity is a crucial sensory characteristic that reflects the ability of a sensor to detect subtle changes in the analyte concentration—in this case, pH. It is essentially a measure of the responsiveness and precision of a sensing device, which provides valuable insights into its performance. The sensitivity (*S*) of a sensor is defined as the slope of the linear range of the sigmoid response curve. In this study, sensitivity was calculated by employing linear regression analysis on the data points within the linear range of approximately ±1 pH units about the p$K_{a}$. The resulting slope represents the rate of change in the sensor’s output with respect to a pH unit change in the analyte concentration.

**Figure 8:**
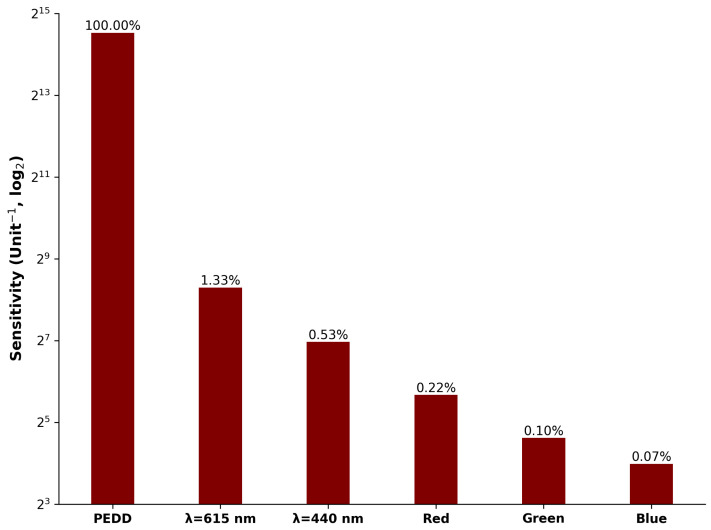
Ranking of the device’s sensitivity from most to least favourable (**left** to **right**). Presented values are relative to the PEDD sensitivity device.

Figure 8 visually presents the sensitivity results for the spectra (at λ=440 nm and λ=615 nm), PEDD, and the camera (Red, Green, and Blue Channels)—see also Table 1 (Band 4). The devices are ordered from left to right based on their sensitivity, with the most sensitive device on the left. The values above each bar show relative sensitivities to the best-performing approach (PEDD). Upon analysis of the data, it is evident that the PEDD approach exhibits the highest sensitivity, followed by the spectrophotometer, while the imaging approach lags behind. This observation is crucial in determining the suitability of these devices for applications requiring high sensitivity in pH measurements. The relative difference in performance is significant when examining the performance of the PEDD approach, with the next closest performing being the spectrophotometer (615 nm) at 33%.

#### 3.4.4. Limit of Detection

The limit of detection (LOD) is a critical parameter in evaluating the performance of a sensing device, representing the lowest concentration of an analyte that can be reliably distinguished from the background noise. Achieving a low LOD is essential for enhancing the sensor’s capability to detect trace amounts of the target analyte. In this study, the LOD was calculated as $3\times \sigma_{blank}/m$, where $\sigma_{blank}$ is the standard deviation of the blank signal and *m* is the slope, following a common approach in analytical chemistry, as used in previous works [23,43,55]. Although the full pH response of BCG is sigmoidal, the LOD was calculated using the linear dynamic region of the response curve (typically spanning the steepest section between two plateaus). This ensured that the LOD estimation adhered to the assumption of linearity in the applied formula and provided a meaningful comparative metric.

Figure 3 presents the absorbance values at 440 nm (left) and 615 nm (right) that were extracted from Figure 2 and plotted as a function of the measured pH. The black squares represent the average across the three measurements, with the error bars as the standard deviation. The red line represents an excellent sigmoid fit to the data points with R^2^ = 0.997, and the optimised parameters are listed in Table 1 (Band 2). In order to estimate the p$K_{a}$ of the dye, the derivative of the fitted model was calculated using Equation (2) and shown in the respective sub-figures as the blue lines, with its peak determined to represent the point of maximum transition between the acid and base form. The p$K_{a}$s were estimated at 4.504 (440 nm) and 4.535 (615 nm), which slightly differ from the accepted value of 4.74 at room temperature [53] by 0.236 and 0.205, respectively. This is a very close approximation and in good agreement with previous reports in the literature, e.g., by O’Toole et al. [45], who estimated the p$K_{a}$ of BCG via PEDD to be 4.5.

#### 3.4.5. Ranking

In the preceding sections, we delved into specific sensing characteristics, which offered a detailed exploration of each approach’s performance. In this section, we seek a comprehensive means to compare these approaches overall. To achieve this, we applied equal weighting to each sensory characteristic, recognising that scholars may adjust these weights based on their specific requirements for different applications.

**Figure 9:**
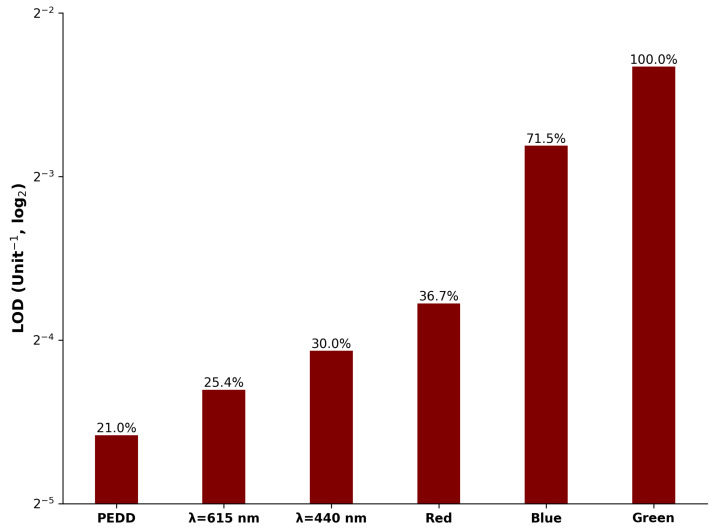
Limit of detection of the devices ranked from left to right in terms of best to least favourite LOD. The presented values above the bars show their relative performance against the least favourite performer (the imaging device’s Green Channel).

Table 2 encapsulates the ranking of each approach, corresponding to the sensory characteristics explored in previous analyses—e.g., refer to Figure 9 for LOD scores. The scores were summed and ranked according to the highest total score. This method, though straightforward, provides a valuable overview of the overall performance of each approach under equal weighting. The results reveal that the PEDD secured the top position (24 points), with the spectrophotometer closely following (average score of 17). In contrast, the imaging approach, despite variations in individual characteristics, averaged 8.7. This ranking demonstrates the PEDD’s superior overall performance when considering the selected sensory characteristics.

It is important to note that the equal weighting applied in this analysis serves as a baseline, and scholars are encouraged to tailor the weights to align with their specific application needs. This flexibility ensures a more nuanced and context-specific approach when choosing the most suitable sensing method. In summary, the ranking chart offers a holistic perspective, aiding scholars in making informed decisions based on their unique priorities and preferences for different bio/chemical sensing applications.

#### 3.4.6. Experimental Error Analysis and Limitations

All experiments were conducted under controlled conditions in a certified analytical chemistry laboratory with stable temperature and humidity, ensuring consistency with standard practices reported in the bio/chemical sensing literature. While this study did not undertake a full error propagation analysis, the following points outline the key considerations taken to ensure measurement integrity:

- Spectrophotometer measurements relied on manufacturer-calibrated devices. Their variability is primarily system-dependent and consistent with other spectrophotometric studies. Additionally, we conducted a baseline while performing measurements in triplicate.
- Imaging-based readings were taken with commercially available RGB cameras, where reflections from cuvette walls were identified as a potential error source. To mitigate this, only the central image region was analysed, as described in the methods.
- Photometric PEDD-based sensing is inherently sensitive to electromagnetic interference due to the low-current photodiode design. As such, all PEDD measurements were conducted within a grounded Faraday cage to minimise electrical noise—particularly relevant for field or industrial deployment scenarios.

All measurements were conducted in triplicate, with the results reported as means accompanied by standard deviation error bars, which is standard across similar sensing studies. To further support transparency in our assessment of variability, Table 1 (Band 5) includes the Relative Standard Deviation (RSD%) for each sensing channel. These values, ranging from 1.62% to 3.21%, demonstrate high measurement reproducibility.

The aim of this study was to compare three different sensing modalities under consistent experimental conditions. As such, we have aligned our error reporting with accepted norms in the field, providing clear and comparable metrics without introducing inconsistent analytical frameworks.

### 3.5. Further Discussion

#### 3.5.1. Photosensor

Traditional spectrophotometers employ fundamental photosensors, such as CCD or high-end photodiodes, responding galvanometrically. These sensors are followed by trans-impedance amplifiers and hardware filtering, with the exact components often undisclosed due to commercial confidentiality. A digital-to-analog (DAC) sub-system is often necessary for achieving high-quality readings at all wavelengths. Cameras utilise similar photosensors and conditioning circuitry, albeit they are typically less sophisticated. The digitisation of analog signals in cameras is usually at 8-bit resolution (0–255), balancing practical compatibility with human vision and compression needs.

While imaging devices may not match spectrophotometers in performance, their shared measurement principle helps explain why the camera in this study offers good operational responses compared to the spectrophotometer. For instance, the camera provides a good estimation of the p$K_{a}$, while the spectrophotometer excels in other sensory characteristics, such as sensitivity and the limit of detection.

The LED in the PEDD operates differently, as explained in previous work [41]. This variance in operational principle may account for the significantly superior sensory characteristics of the PEDD compared to both the spectrophotometer and camera. Previous comparisons for turbidity measurements [23] revealed a lower limit of detection by a factor of 9.3 and a higher sensitivity factor of 117. This increased performance is further evident in this study. The temporal-based bit depth on the discharge counter, which differs from traditional galvanometric responses, likely contributes to the enhanced performance of the LED.

#### 3.5.2. Cost

The high-level spectrophotometer design mentioned above, with a diffracting grating and controlled movement for detecting absorption at all wavelengths, explains why spectrophotometers typically cost thousands of pounds or dollars. In contrast, cameras, with their wide-band photosensors capturing the full visible range, are more affordable, costing hundreds or more, and are also commonly integrated into smartphones. LEDs present an even more cost-effective alternative, with costs in the cent range, making them attractive for scalability.

#### 3.5.3. Implementation

Implementation costs also vary among these approaches. A spectrophotometer demands specialised/high-end components and electronic engineering. Cameras, being miniaturised devices, require software engineering skills for computer vision requirements. LEDs necessitate a more precise level of skill and understanding of this newer method. In terms of both hardware and software requirements, several factors must be considered.

Firstly, the spectrophotometer and LED essentially provide zero-dimensional spatial measurement when compared to the 2D nature of the imaging devices. This typically requires full light control/encapsulation from the surrounding environment, whereas there have been some reports on the ability for imaging devices to operate well under varying lighting conditions [22,27,56]. This is likely due to the three spectral measurements (Red, Green, and Blue) captured together and using those for light-independent analysis, e.g., via the HSV colour space [57]. Imaging devices can also perform parallel reference measurements, as reported by Curto [58] or Fay [59], offering a more efficient, robust, and temporal approach.

Secondly, with the need to address the per-measurement cost—as pointed out by Murray [14] and discussed in the introduction—an important aspect of this is the hardware required to transfer the information provided by the optical transducer to quantitative values for analysis. The PEDD approach is unique in this respect insofar that its resolution is based on discharge time, and the DAC uses a single microcontroller IO. Each microcontroller/processor is equipped with digital IOs capable of performing a PEDD measurement allowing for optimum scalability. Essentially, this approach does not enter the ADC bit-depth race that may be required for the spectrophotometer/imaging, whereby microcontrollers/DAQ systems can range from 8-bit (e.g., PIC16F1824, Microchip Technology, Chandler, Arizona, USA), to 32-bit (e.g., ADS1285, Texas Instruments, Dallas, Texas, USA), with the cost scaling proportionally and therefore negatively affecting scalability. The PEDD, on the other hand, is more adaptable with a simple counter dictating the bit depth, which can be altered in software. Additionally, the emitter intensity can be reduced on the PEDD, leading to a higher resolution due to the inverse measurement principle.

Finally, it must be noted that software elements are another necessary resource required by sensing elements. With the increasing trend towards the use of machine learning (ML) or Artificial Intelligence (AI) for sensory data [43], the allowances for software resources have become essential. This is more of a challenge for imaging sensing, as spatial depth (megapixels) is increasing along with the processing steps, e.g., native photosensors to CIE XYZ, conversion to sRGB (or others), segmentation algorithms, reference systems, and analysis. While spectrophotometers are generally commercial in nature, they are often supplied with customised applications and firmware for operational purposes.

#### 3.5.4. Specificity vs. Generalisation

Spectrophotometers and LEDs typically operate by choosing a specific wavelength for analysis, allowing for high precision in measurements. The PEDD, with its limited waveband, lacks versatility for sensing other colourimetric indicators without LED component alterations. In contrast, imaging devices, equipped with three widespread photodetectors, can detect the visible spectrum, sacrificing some precision for the ability to analyse multiple dyes without hardware modifications.

#### 3.5.5. Imaging

The camera used in this study, representing a common output for most imaging devices on the market, was 24-bit, i.e., 3×3×3 8-bit. While some devices capture at higher bit rates and different colour spaces offer increased bit depths, RGB remains the most prevalent format. As imaging technology advances, future studies may yield sensory characteristics approaching those of a spectrophotometer. In terms of bit depth, however, this may be unlikely, as 8-bit is the accepted standard and may not increase, as the ultimate goal is to capture enough to reproduce human vision; otherwise, the superfluous information would be a waste in terms of data transmission/storage.

#### 3.5.6. Bio/Chemical Sensing

The integration of optical and colourimetric sensing has significantly propelled advancements in bio/chemical sensing, offering rapid, sensitive, and cost-effective detection methods. Examples include point-of-care virus detection [60,61,62], environmental analysis [15,30,55,63], and food safety [64,65,66,67,68,69,70].

In terms of the sensing technology used in this domain, there again have been the primary three approaches outlined in this work—namely, imaging [71,72,73], LED photometry [74,75,76], and the spectrophotometer [77,78,79]. While it is well recognised that the spectrophotometer is the de facto standard, it must be noted that in bio/chemical sensing, minute quantities are required and therefore necessitate a highly sensitive detector. The findings in this study show that the LED in PEDD discharge mode outperformed the spectrophotometer, showing higher sensitivity, resolution, and a better LOD. Imaging may offer advantages in areas such as determining live-dead staining where there are two clear binary classifications and colours.

Overall, where a low-power and integrated portable system is required, the LED appears to be the best option. However, in domains where personalised checks are to take place and the colourimetric reagent is optimised for naked eye detection, the imaging approach can provide a faster means, given the ubiquitous nature of this technology. Another realm where the imaging and spectrophotometer have clear advantages is often where multiple reagents/targets are in use. Given that the LED system is focused with one wavelength, it requires arrays and/or replacement for an additional target, whereas the imaging/spectrophotometer spans the full visible range.

### 3.6. Applicability of Bromocresol Green and Methodological Versatility

Bromocresol green (BCG) was selected for this study due to its well-characterised pH transition range, strong molar absorptivity, and spectral overlap with the RGB channels, making it suitable for evaluating the performance of various optical sensing modalities. BCG is widely used in industrial applications, including environmental monitoring [80,81] and clinical diagnostics [82,83], due to its reliable colourimetric response in acidic to neutral pH conditions.

In biosensing applications, the pH range of interest typically spans from 4 to 9, encompassing physiological conditions such as blood (pH 7.4) [84], saliva (pH 6.2–7.6) [85], and urine (pH 4.5–8.0) [86]. While BCG’s range does not cover the entire spectrum of these applications, it serves as a representative model for acidic to neutral conditions, which are pertinent in various industrial contexts.

Optical sensors, including photometric LED devices (PEDDs), imaging systems, and spectrophotometers, have been extensively utilised in industrial settings for monitoring chemical processes, environmental parameters, and quality control. For instance, PEDDs have demonstrated efficacy in detecting specific analytes in manufacturing processes, while imaging systems are employed for rapid assessment of product quality. Spectrophotometers, known for their precision, are standard tools in laboratories for substance quantification.

The methodology presented in this study provides a foundational platform for evaluating and comparing these optical sensing techniques. By establishing a standardised approach using BCG, we enable future research to adapt this framework for other indicators and analytes relevant to industrial applications, such as ammonia detection in wastewater treatment or glucose monitoring in fermentation processes. This adaptability underscores the versatility and scalability of the proposed methods, aligning with the objectives of advancing optical sensor technologies for diverse industrial applications.

#### 3.6.1. Microfluidics

Related to the field of bio/chemical sensing, especially for personal and environmental diagnostic purposes, microfluidics [19,87,88,89] as a transformative technology has offered numerous advantages in terms of miniaturisation, integration, automation, and sensitivity. It is common to use cameras for imaging in microfluidic assays that require quantitative colour readouts [90,91,92,93,94]; however, uncertainty and inconsistency from the fluctuation in lighting conditions could compromise the sensitivity in colourimetric readings captured via camera-based methods. The LED photometry approach mentioned in the work had a positive impact on quantitative measurements from colourimetric tests [95] in terms of resolution, accuracy, sensitivity, and the limit of detection. While imaging technology has seen some promise in colour constancy, as discussed in Section 3.5.3, the LED—much like the spectrophotometer—requires external light encapsulation. Considering, however, that optical chambers in microfluidic channel design can be on the micro level [96,97,98], this presents a challenge in terms of the path length. As a result, one would require a highly sensitive detector for successful operation. Based on the comparative findings in this study, the PEDD offers the best approach in terms of sensory characteristics, particularly sensitivity and resolution.

#### 3.6.2. Industrial Relevance and the Need for Comparative Evaluation

In many industrial contexts, stakeholders are faced with the decision of adopting a monitoring system requiring optical sensing capabilities. Each method provided herein offers distinct advantages depending on factors such as sensitivity requirements, cost constraints, integration ease, and environmental robustness. However, the literature is often standalone and lacks direct standardised comparisons between these approaches under controlled conditions to better inform their choice.

This study fills that gap by providing a controlled, side-by-side comparison of three widely used optical sensing modalities. While no individual extension of these sensing principles is offered, the aim was to evaluate existing and established technologies/approaches fairly and transparently. Introducing individual innovation within the tested methods would have undermined the comparative intent of the work and may be out of step with the established methods in the literature.

The framework established here provides a foundation for future investigations across other colorimetric reagents and application domains. It supports the industry in making more informed decisions about sensing technologies and offers a reference point for evaluating performance tradeoffs in real-world scenarios.

Future research may build upon this comparative platform by applying it to other analytes or environmental parameters or by evaluating novel sensor materials within the same experimental structure. This will ensure that both innovation and comparability can be achieved in tandem.

## 4. Conclusions

This study presented a unified comparison of three optical sensing approaches for colourimetric bio/chemical determination using a pH indicator: (i) laboratory-grade spectrophotometry ($\lambda_{440}$, $\lambda_{615}$), (ii) portable imaging (sRGB via camera), and (iii) low-power, low-cost LED photometry (Paired Emitter–Detector Diode, PEDD). Among these, the PEDD approach consistently demonstrated the highest performance across all measured sensory characteristics.

A ratiometric analysis of key metrics (LED : spectrophotometer : imaging) revealed that the LED method excelled in measurement range (100 : 6.1 : 0.39), dynamic range (100 : 0.68 : 0.15), accuracy in p$K_{a}$ estimation (2.6 : 4.3 : 4.1%RE), sensitivity (100 : 0.93 : 0.13), and limit of detection (1 : 1.32 : 9.91) results. These findings reinforce the viability of the PEDD method as a high-performance, cost-effective alternative to conventional laboratory-based or portable imaging solutions.

Beyond academic benchmarking, this comparative evaluation offers practical guidance for industrial and field-based applications where cost, scalability, and operational simplicity are key drivers. The PEDD approach supports decentralised sensing, autonomous deployment, and integration into scalable monitoring systems—critical for sectors such as environmental diagnostics, industrial safety, pharmaceutical testing, and health monitoring.

In summary, the results provide a roadmap for selecting suitable optical sensing strategies based on performance and application needs, with the PEDD method emerging as a compelling candidate for widespread, real-world adoption.

## Figures and Tables

**Table 1:** Comparison table of all approaches. Reference figure and estimated p$K_{a}$ (Band 1), optimised fitting parameters when applying Equation (1) to the datasets of the spectra (Band 2), device specifics (Band 3), sensory characteristics (Band 4). All values have been rounded to two decimal places. Sensory characteristics are expressed in device-specific units (e.g., intensity counts) for comparative purposes and are not directly presented in common or SI units.

Parameter	Spectra (λ=440nm)	Spectra (λ=615nm)	PEDD	Red Ch.	Green Ch.	Blue Ch.
Figure	Figure 3 (Left)	Figure 3 (Right)	Figure 4	Figure 5 (Top)	Figure 5 (Middle)	Figure 5 (Bottom)
p$K_{a}$	4.54	4.5	4.87	4.36	4.5	4.94
y_0_	0.27	0.05	63,116	191.26	171.86	96.76
y_1_	0.05	0.58	7889.3	46.7	109.48	131.22
x_0_	4.5	4.54	4.87	4.36	4.5	4.94
dx	0.27	0.29	0.5	0.48	0.48	0.41
R^2^	1	1	1	0.99	0.98	0.92
Device FSD	4.000	4.000	65,535	255	255	255
Unit Step	0.001	0.001	1	1	1	1
Increments	4000	4000	65,535	255	255	255
Dynamic Range	0.22	0.52	55,226.7	144.56	62.37	34.47
Resolution	222	524	55,226.7	144.56	62.37	34.47
Sensitivity	125.29	314.7	23,657.06	51	24.57	15.86
LOD	0.06	0.05	0.04	0.07	0.2	0.14
RSD (%)	3.11	3.08	3.07	3.21	1.62	1.71

**Table 2:** Ranking of all 6 approaches based on the comparisons of 4 categories: resolution, accuracy, sensitivity, and limit of detection. Top ranked per category score 6, while the lowest scores 1.

Approach	Resolution	${\mathrm{Accuracy}}_{{pK}_{a}}$	Sensitivity	LOD	Score	Ranked
λ = 440 nm	4	4	4	4	16	3rd
λ = 615 nm	5	3	5	5	18	2nd
PEDD	6	6	6	6	24	1st
Red	3	1	3	3	10	4th
Green	2	2	2	2	8	5th=
Blue	1	5	1	1	8	5th=

## Data Availability

The original contributions presented in this study are included in the article. Further inquiries can be directed to the corresponding author.

## Abbreviations

The following abbreviations are used in this manuscript:

PEDD	Paired Emitter–Detector Diode
LED	Light-Emitting Diode
IoT	Internet of Things
BCG	Bromocresol green
CAD	Computer-Aided Design
nm	nanometer
RGB	red, green, and blue
CIE	Commission Internationale de l’éclairage
HCl	Hydrochloric acid
KOH	Potassium hydroxide
mg	milligram
mL	millilitre
mm	millimetre
UV-Vis	Untraviolet–Visible
CAD	Computer-Aided Design
ABS	Acrylonitrile Butadiene Styrene
GND	Ground
IO	Input/Optpuut
ROI	Region of Interest
p$K_{a}$	Acid Dissociation Constant
FSD	Full-Scale Deflection
%RE	Percentage Relative Error
